# Proteins and Their Interacting Partners: An Introduction to Protein–Ligand Binding Site Prediction Methods

**DOI:** 10.3390/ijms161226202

**Published:** 2015-12-15

**Authors:** Daniel Barry Roche, Danielle Allison Brackenridge, Liam James McGuffin

**Affiliations:** 1 Institut de Biologie Computationnelle, LIRMM, CNRS, Université de Montpellier, Montpellier 34095, France; 2 Centre de Recherche de Biochimie Macromoléculaire, CNRS-UMR 5237, Montpellier 34293, France; 3 School of Biological Sciences, University of Reading, Reading RG6 6AS, UK; d.a.brackenridge@pgr.reading.ac.uk (D.A.B.); l.j.mcguffin@reading.ac.uk (L.J.M.)

**Keywords:** protein–ligand binding site prediction, protein function prediction, binding-site residue prediction, biochemical functional elucidation, sequence-based function prediction, structure-based function prediction, biological and biochemical role of enzymes, gene Ontology, enzyme commission numbers

## Abstract

Elucidating the biological and biochemical roles of proteins, and subsequently determining their interacting partners, can be difficult and time consuming using *in vitro* and/or *in vivo* methods, and consequently the majority of newly sequenced proteins will have unknown structures and functions. However, *in silico* methods for predicting protein–ligand binding sites and protein biochemical functions offer an alternative practical solution. The characterisation of protein–ligand binding sites is essential for investigating new functional roles, which can impact the major biological research spheres of health, food, and energy security. In this review we discuss the role *in silico* methods play in 3D modelling of protein–ligand binding sites, along with their role in predicting biochemical functionality. In addition, we describe in detail some of the key alternative *in silico* prediction approaches that are available, as well as discussing the Critical Assessment of Techniques for Protein Structure Prediction (CASP) and the Continuous Automated Model EvaluatiOn (CAMEO) projects, and their impact on developments in the field. Furthermore, we discuss the importance of protein function prediction methods for tackling 21st century problems.

## 1. Introduction

Proteins are essential molecules involved in a wide variety of essential intra- and inter-cellular activities. These activities include, but are not limited to: maintaining cellular defences, enzymatic catalysis, metabolism and catabolism, maintenance of the structural integrity of cells, and signalling within and between cells. Furthermore, protein–ligand interactions are essential for biochemical functionality and are implicated in all biochemical roles, in all kingdoms of life. Hence, studying protein–ligand binding sites and their associated residues, is an important step in the functional elucidation of proteins involved in these cellular processes [1,2,3,4].

**Figure 1:**
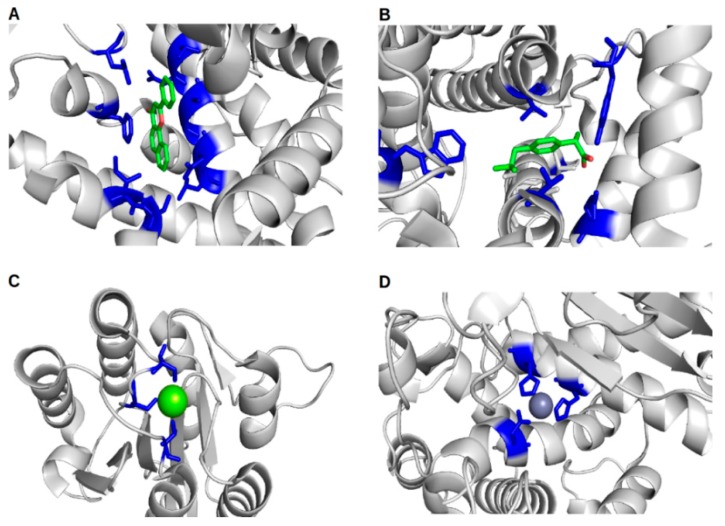
Examples of protein–ligand interactions, focusing on the ligand binding site. Proteins are shown in cartoon form and coloured in light grey, with binding site residues shown as blue sticks, and ligands shown as sticks or spheres coloured by element; (**A**) The Human cytochrome P450 1A1 protein (PDB ID 4i8v) bound to the drug *N*-Benzylformamide; (**B**) Cyclooxygenase-2 (PDB ID 4ph9) from *Mus musculus* bound to the drug Ibuprofen; (**C**) The *Plasmodium vivax* TRAP protein (PDB ID 4hqo, CASP ID T0686) bound to magnesium and; (**D**) The aminopeptidase N family protein Q5QTY1 (PDB ID 4fgm, CASP ID T0726) from *Idiomarina loihiensis* bound to zinc (a cofactor).

Understanding protein–ligand interactions in the context of protein–ligand binding sites and ligand binding site residues is important for fully understanding cellular mechanisms, and is critical for understanding responses to drugs. Methods for the prediction of protein–ligand binding sites, which are detailed in the following section, can greatly enhance our understanding of the molecular mechanisms involved in many research spheres, helping us tackle numerous 21st century problems. The effects of protein–ligand binding are transient, but this knowledge can be exploited for the treatment of human and animal diseases, in addition to impacting food security research, examples of which are highlighted in Figure 1 and discussed in Section 6.

We begin by briefly highlighting some key protein–ligand interactions from a biomedical perspective. In Figure 1 we focus on four examples of proteins bound to diverse types of ligands, which are important in health and disease. This includes Cytochrome P450 bound to the drug *N*-Benzylformamide (Figure 1A—PDB ID 4i8v). The enzyme Cytochrome P450 has an essential role in the electron transfer chain, and is therefore ubiquitous in all kingdoms of life [5]. The human Cytochrome P450 (CYP1A1) is known to play a role in the biotransformation of polycyclic aromatic hydrocarbons into carcinogens [6]. In addition, CYP1A1 (PDB ID 4i8v) is responsible for the metabolism of theophylline [7], a drug used to provide symptomatic relief from asthma. Cyclooxygenase-2 from *Mus musculus*, which is involved in the biosynthesis of prostaglandins, is a target of non-steroidal anti-inflammatory drugs such as Ibuprofen (Figure 1B). The *Plasmodium vivax* TRAP protein, bound to magnesium, is involved in phosphate ester hydrolysis (Figure 1C). Finally, Figure 1D shows the protein–ligand binding site of the aminopeptidase N family protein Q5QTY1, from *Idiomarina loihiensis* bound to zinc (its cofactor), which can be used as a biomarker to detect kidney damage.

This review aims to provide an overview of the variety of different methodologies available for the prediction of protein–ligand binding sites and their associated binding site residues. Here we will focus on computational methods developed in the last six years, since the inclusion of the function prediction (FN) category in the Critical Assessment of Techniques for Protein Structure Prediction (CASP) competition [8]. For methods developed before 2010, please refer to the review by Kaufmann and Karypis [9]. Furthermore, molecular docking methods are beyond the scope of this review, which have been recently reviewed by Yuriev *et al.* [10]. In this review, the term ligand is used to refer to molecules capable of binding to a protein, such as metal ions, small organic (e.g., ATP) and inorganic compounds (e.g., NH_4_), peptides, and DNA/RNA; not large macromolecules such as proteins.

## 2. *In Silico* Methods for the Prediction of Protein–Ligand Binding Sites and Their Associated Binding Site Residues

In recent years, a large number of methods have been developed for the prediction of protein function and protein–ligand binding sites. In this review, we discuss methods for the prediction of protein–ligand binding sites and their associated binding site residues. These methods can be broadly divided into sequence-based methods and structure-based methods.

### 2.1. Sequence-Based Methods

Sequence-based methods that predict protein–ligand binding sites and their interacting ligand-binding site residues are those that use information from evolutionary conservation and/or sequence similarity of homologous proteins. These methods can be broadly categorised into methods that utilize machine learning (Multi-RELIEF [11], TargetS [12], LigandRF [13], and OMSL [14]), methods that utilize only position-specific scoring matrices or PSSMs (INTREPID [15], DISCERN [16], ConSurf [17], and ConFunc [18]) and graph-based methods such as Conditional Random Field (CRF) [19]. The advent of including machine learning-based strategies into sequence-based methods has resulted in improved method sensitivity. Machine learning is applied to PSSMs or multiple sequence alignment-based properties using various alternative strategies, examples of which will now be discussed. 

Many of the sequence-based methods, such as Multi-RELIEF [11], deploy machine learning methods to directly interpret multiple sequence alignment profiles. Multi-RELIEF works by estimating the functional specificity of residues from a multiple sequence alignment using local conservation properties. This method uses a machine learning technique called RELIEF [20] for feature selection and weighting, using a binary classification to discriminate features from two classes. A residue’s local specificity is determined by comparing the sequence with the closest homologue in each of the two classes (same class and opposite class), using global sequence identity to find the nearest neighbour sequence. If a residue has high local specificity to one pair of classes, it is labelled as relevant. Furthermore, global sequence similarity is considered while scoring each residue locally [11]. This results in the prediction of residues comprising a putative ligand binding site.

In contrast, LigandRFs [13] uses a random forest-based algorithm to predict protein–ligand binding site residues. LigandRFs extracts 544 amino acid properties from the AAindex database [21], which are then compared using the Matthews correlation coefficient. Each of the 544 properties are ranked in relation to the number of their related properties. The properties are filtered to remove all properties related to the top property; this removes redundant properties, which do not add any new information. This process is continued through the list until 34 properties remain. These properties relate to specific features crucial for determining putative binding site residues. The properties are then applied over a seven residue sliding window of a PSI-BLAST [22] profile. A 1 × 238 vector is used to represent the 34 amino acid properties for each seven residue window. A random forest is then utilized to learn the relationship between the large vector and the binding or non-binding residue properties [13].

TargetS [12] is another machine learning-based method, but in contrast to other methods, it utilizes secondary structure-based features in addition to sequence and PSSM-based features. Currently, TargetS can predict ligand-binding sites for proteins that bind to nucleotides, metal ions, DNA, and heme. The algorithm incorporates: protein conservation from a PSI-BLAST [22] PSSM searching SwissProt [23], secondary structure features determined from the PSIPRED algorithm [24], along with ligand-binding propensity of residues for each amino acid and each ligand category (nucleotides, metal ions, DNA, and heme). These properties are subsequently combined using a support vector machine (SVM) to predict ligand-binding site residues.

### 2.2. Structure-Based Methods

Structure-based methods are those that exploit information from 3D atomic coordinates (either predicted from sequence or derived from experiments). These methods either predict the location of the ligand binding site and/or the putative ligand binding site residues. Such methods can be further sub-categorised into: 1. Geometric-based methods (FINDSITE [25], LigDig [26], LISE [27], PatchSurfer2.0 [28], Surflex-PSIM [29], EvolutionaryTrace [30], PRANK [31], a Two-dimensional replica-exchange method [32], FMO-RESP [33], MapReduce approach [34], TIFP [35], ProGolem [36], a Chemogenomics approach [37], ProPose [38], FunFHMMer [39], mFASD [40], ProBis [41,42], and CavBase [43,44]); 2. Energetic methods (SITEHOUND [45], VISCANA [46], SiteComp [47], and FTMap [48]); 3. Miscellaneous methods, which use information from homology or template-based modelling (FunFOLD3 [3,4], COACH [49], COFACTOR [50], GalaxySite [51], GASS [52], VISM-CFA [53], and PLIP [54]), Surface accessibility based methods such as LigSite^CSC^ [55], in addition to Physicochemical properties exploited by Andersson and colleagues [56]. Examples of different methods from each sub-category are now described, in addition to their limitations.

#### 2.2.1. Considerations When Employing Structure-Based Methods

Structure-based methods for prediction of protein–ligand binding sites have a number of limitations, including the following: 1. If a 3D model or experimental structure cannot be obtained, then it is not possible to make a prediction; in such cases the solution is to rely on purely sequence-based methods. 2. If templates with the same fold as the target protein that contain biologically relevant ligands cannot be detected, then it is not possible to make a prediction. 3. Most prediction servers, such as COACH [49] and FunFOLD [3,4,57], utilize in-house structure prediction pipelines to construct models for protein–ligand interaction predictions that may not always produce the best quality model for every target, which may result in over- and under-predicted protein–ligand binding sites. Nevertheless, despite these shortcomings, prediction methods are constantly under development and improvements can be gauged via the rigorous independent blind assessment scoring, described in Section 3.

#### 2.2.2. Geometric Methods

FINDSITE [25] combines evolutionary and structural information to predict protein function, identifying binding pockets based on binding site similarity between homologous structures. This is undertaken by superposing templates onto the structure of interest and then finding sites where ligands overlap. These results are then used to determine putative binding pockets and then identifying the geometric centre of each pocket [25]. 

Similarly, LigDig [26] is another geometric method, but uses a ligand-centric approach, rather than the traditional protein-centric approach to detect ligand-binding pockets in proteins. LigDig utilizes a variety of information from ChEBI [58], PubChem, PDB [59], UniProt [23], and KEGG [60], combined via a graph-based network to locate similar ligands along with their potential binding partners. The method is available as a webserver and also uses text-based searches to find proteins that may bind to a particular ligand of interest [26]. This results in the prediction of putative protein–ligand binding sites.

In contrast to FINDSITE, LigDig, and the majority of geometric-based approaches, LISE [27] is an algorithm that utilizes a novel concept of binding site-enriched protein triangles in order to predict protein–ligand binding site locations. LISE uses ideas developed in a previous method, called MotifScore [61], that determined motifs in a protein–ligand interaction database, composed of 6276 protein–ligand structures. The motifs contain the interactions between three atoms of a protein and two atoms of a ligand. Thus, the three protein atoms of these motifs compose the “protein triangles”. An additional step is to encapsulate the protein into a 3D grid of 1 Å size steps. Each vertex in this grid is then labelled as occupied or empty (with a 2.7 Å distance cutoff). For each empty grid point, a grid point score is calculated, which equals the sum of the triangle scores. A large sphere of 11Å is then centred on each empty vertex, and for each sphere, a sphere score is calculated, which is based on the sum of the grid point scores for all empty grid points within the sphere. The sphere with the highest score is determined as the putative ligand binding site [27].

#### 2.2.3. Energetic Methods

SITEHOUND [45] is a widely used energetic method for the prediction of protein–ligand binding sites, which utilizes a chemical probe to explore the surface of the protein structure, determining regions that may have optimum energy for binding. SITEHOUND uses two different chemical probes: a carbon probe to identify drug-like binding sites, and a phosphate probe to locate binding sites for ligands having a phosphate group. Affinity maps or molecular interaction fields are then used to describe the interaction of each probe with the protein surface. These affinity maps are subsequently filtered to remove unfavourable interaction energies. The next step is to utilize agglomerative hierarchical clustering to cluster the remaining interaction points based on their spatial proximity. These clustered points are ranked by total interaction energy and result in a list of potential ligand-binding pocket locations [45].

#### 2.2.4. Miscellaneous Methods

A recent review by Petrey *et al*. [62] highlights the essential need for template-based 3D modelling methods in the prediction of protein function [62]. The majority of these methods predict putative protein–ligand binding sites and ligand binding site residues, while some methods additionally predict Enzyme Commission Numbers (EC) and Gene Ontology (GO) terms. We have developed a number of versions of a template-based method, called FunFOLD [3,4,57], which starts with a 3D model of the target protein predicted from sequence, for example using the IntFOLD server [63,64]. Each version of the algorithm has worked on the assumption that proteins with the same fold that bind to similar biologically relevant ligands are likely to have similar binding sites. The latest FunFOLD3 pipeline is composed of updated versions of two main algorithms, FunFOLD [4] and FunFOLDQA [1], and it produces output comprising predicted EC and GO terms, ligand-binding site residues, putative ligands, binding site quality scores, and per-atom *p*-values to comply with the CAMEO-LB format [65]. 

FunFOLD firstly superposes, using TM-align [66], a list of structural templates containing biologically relevant ligands (determined using the BioLip database [67]) onto the target 3D model. Template-model superpositions with a TM-score ≥ 0.4 are retained. The next step is to superimpose all retained templates onto the target model and assign ligands from the template files into clusters using agglomerative hierarchical clustering. The identified ligand clusters are located at the potential ligand-binding sites. Ligands are determined to be components of a cluster if the contact distance is less than or equal to 0.5 Å plus the Van der Waal radii of the contacting atoms. The putative ligand-binding site containing the largest ligand cluster is determined to be the most probable ligand-binding site of the protein. The identification of the putative ligand-binding site residues is carried out via a residue voting method [3,4].

The next component of the FunFOLD3 pipeline is the FunFOLDQA algorithm [1], which evaluates the quality of FunFOLD predictions, subsequently producing a set of confidence scores. The algorithm outputs scores for five sequence- and structure-based features that are combined using a neural network, outputting predicted Binding-site Distance Test (BDT) [68] and Matthews Correlation Coefficient (MCC) [69] scores. The FunFOLD3 [57] pipeline additionally outputs a set of per-residue binding probability scores to comply with the CAMEO-LB format [65]. Furthermore, the FunFOLD3 method outputs a putative ligand binding site, putative ligand binding site residues, putative ligands that may bind to the target protein, along with predicted EC and GO [70,71] terms (see Section 4) for each target protein [3,4].

The COACH [49] method is similar to FunFOLD and is one of the most accurate ligand-binding site prediction methods that utilizes both sequence and structural homology in the prediction pipeline. The structure component (TM-SITE) of the pipeline firstly locates putative ligand-binding pockets using ConCavity [72]. TM-SITE then uses fifteen residues within the binding pocket structure to search against the BioLip database to find structures containing similar binding pockets, in addition to searching for similar structures (using TM-align [66]) to the target protein containing biologically relevant ligands within BioLip [67]. All templates and sub-structural templates are superposed onto the target and scored based on empirically determined cutoffs. Ligand binding site residues are then determined using a similar strategy to FunFOLD [4], but using average linkage clustering and assigning a confidence score to each predicted ligand binding site residue. The sequence component of the algorithm, S-SITE, uses residue conservation of sequence profiles to predict ligand binding site residues, subsequently scoring the confidence of each predicted binding site residue. COACH then uses a consensus of predictions, combining the results from TM-SITE and S-SITE along with COFACTOR [50], FINDSITE [25], and ConCavity [72]. Similar to FunFOLD3, COACH predicts a putative ligand binding site, putative ligand binding site residues, putative ligands that may bind to the target protein, along with predicted EC and GO terms for each target protein. 

A somewhat alternative approach to that of FunFOLD and COACH is used by GASS [52]. GASS (Genetic Active Site Search) is developed by Izidoro *et al.* [52], who have employed a genetic algorithm to predict ligand binding site residues for putative enzymes. Their method takes a list of templates from the CSA [73] with predefined binding site residues. They then simulate evolutionary effects (crossover and mutations) over this population of templates, according to predefined mutational probabilities, for a specific number of user-defined generations. The resultant binding site residue predictions are then assessed using a fitness function, which ranks individual sets of predictions. The fitness function is similar to an RMSD (root-mean-square deviation) for the ligand binding site residues, with the main difference being that the square distance of the results is not averaged [74].

Several structure-based methods that exploit surface accessibility have also been developed, such as LIGSITE^csc^ [55]. LIGSITE^csc^ uses the Connolly surface in its ligand binding site prediction protocol. The first step of the protocol is to encapsulate the protein structure into a 3D grid of 1 Å steps. In the second step of the protocol, each point in the grid is labelled as either protein, surface, or solvent. In the third step, the Connolly algorithm is utilized to calculate the solvent-excluded surface. In the fourth step, surface-solvent-surface events are then determined. In the fifth step, if the surface-solvent-surface events in a grid exceed a minimum threshold, set to six grid locations, this is determined to be a pocket. Each pocket cluster is then ranked in relation to the number of grid points within the cluster. The top three pockets are then retained. In the final step, the top three pockets are re-ranked in relation to the conservation of pocket surface residues [55]. 

Further structure-based methods have used physiochemical properties to determine ligand binding cavities. For example, the method by Andersson *et al.* (2010, [56]) works initially by identifying solvent accessible patches. In the second step, data is collected from each patch based on 408 surface descriptors, divided into eight categories. These descriptors include neighbouring amino acids, secondary structure, polarity of adjacent amino acids, close hydrogen bond donors and acceptors, electrostatic potential, shape, polarity, and flexibility. In the third step, the descriptor results are divided into bins and scaled to be usable for Principal Component Analysis (PCA). In the fourth step, PCA is carried out and the relationships between pockets are analysed. This method produces results for all putative pockets, leaving the user to determine which pocket is the most suitable ligand binding pocket for their particular task [56].

## 3. Methods for the Evaluation of Protein–Ligand Binding Site Residue Predictions

Assessment of protein–ligand binding site residue predictions have been carried out in CASP [8,75,76] and CAMEO [65] using a number of different scores, which include the Matthews Correlation Coefficient (MCC) [69] and the Binding-site Distance Test (BDT) score [68]. The MCC score is a statistical measure that compares observed ligand binding site residues to predictions by assessing the number of residues assigned as true positives, false positives, true negatives, and false negatives. This results in a score between −1 and 1, with scores close to zero representing random predictions and scores close to one representing near perfect predictions. The MCC score may only be a good choice for scoring sequence-based predictions, when no structural information is available, as the MCC score does not consider the 3D nature of the protein within the scoring metric.

To overcome the limitations of the MCC score, we developed the Binding-site Distance Test (BDT score) [68]. The BDT score utilizes the distance in 3D space between a predicted ligand binding site residue and an observed ligand binding site residue in the scoring process. The BDT score has a range from zero to one, where scores close to zero represent random predictions and scores close to one represent near perfect predictions. Predicted ligand binding sites closer in 3D space to the observed ligand binding site are scored higher than ligand binding sites predicted farther from the observed ligand binding site. In the CASP9 and CASP10 FN assessments [75,76], the BDT score was used by the official assessors in addition to the MCC score. Furthermore, the BDT score is used in the CAMEO [65] project as one of the standard assessment metrics.

## 4. Prediction of Enzyme Commission Numbers (EC) and Gene Ontology Terms (GO)

In addition to the determination of protein–ligand binding sites and their associated binding site residues, it is also useful to determine the likely function of a protein. Functionality can be generally assigned using Gene Ontology (GO) terms [70,71], or more specifically for enzymes, using Enzyme Commission numbers (EC).

The Gene Ontology Commission was formed in 2000 [70] to develop a controlled vocabulary for describing genes, as a result of the large increase of sequence data from genomics projects. Gene Ontology (GO) terms, often referred to as a shared vocabulary for genes, comprise over 40,000 terms. GO terms are broadly divided into three categories: cellular components, molecular function (a weak analogy to EC codes), and biological processes, which are further subdivided in a hierarchical graph-like structure. Each protein has the potential to be assigned to multiple GO classes and sub-classes. Moreover, each GO term has a unique serial number, in addition to a textual description [70,71]. 

The Enzyme Commission (EC) was set up in 1956 as part of the International Union of Pure and Applied Chemistry (IUPAC), publishing the first version of EC numbers in 1961. Today, the EC classification is maintained by the Nomenclature Committee of the International Union for Biochemistry and Molecular Biology (NC-IUBMB) and the enzyme list is curated and maintained by the Tipton group at Trinity College Dublin [77]. The list officially classifies enzymes by the overall reactions they catalyse, in order to reduce the ambiguous names enzymes previously acquired. Enzymes are hierarchically classified by four-digit EC numbers. The first number designates the broad classification into: 1. Oxidoreductases; 2. Transferases; 3. Hydrolases; 4. Lyases; 5. Isomerases; and 6. Ligases. The second class usually designates the type of molecule involved in the reaction. The third class designates the type of reaction involved, while the fourth class is essentially a serial number, which has been utilized to differentiate enzymes within the subclasses [77]. 

Recently, a number of methods have been developed specifically to predict GO and EC terms. A large number of these methods have been developed as rapid methods that utilize sequence information only. The majority of methods predict function based on Gene Ontology (GO) terms (which include: INGA [78], EFI-EST [79], SIFTER [80], GEO2Enrichr [81], PANNZER [82], and PILL [83]) with fewer utilizing EC numbers (EFI-EST [79] and DomSign [84]) for functional annotation. Furthermore, a number of structure-based methods for the prediction of protein–ligand binding sites have incorporated methods for predicting GO and EC terms, including COACH [49] and FunFOLD3 [3,4,57] (See Section 2.2.4). However, as these methods build 3D models as part of their prediction pipeline, they are somewhat more computationally intensive than the sequence-only methods. 

The prediction of EC and GO terms, in addition to the prediction of protein–ligand binding sites and their associated ligand binding site residues, further enriches the information that can be gleaned for a particular protein. This highlights the biological need for *in silico* methods in function prediction and rational drug design, contributing to future *in silico*, *in vitro*, and *in vivo* experiments for both biomedical and bioenvironmental research applications.

## 5. CASP, CAFA, and CAMEO—Their Role in Development and Assessment of Protein–Ligand Binding Site Prediction Algorithms

The development of methods for the prediction of protein–ligand binding sites and function prediction has been driven in recent years as a direct result of community wide prediction experiments, such as the Critical Assessment of Techniques for Protein Structure Prediction (CASP) [8,75,76], the Continuous Automated Model EvaluatiOn (CAMEO) project [65], and the Critical Assessment of Function Annotation (CAFA) [85].

Ligand binding site residue prediction was first introduced in CASP8 (as the FN category) [8], with the concept then involving the prediction of putative ligand binding site residues, which may functionally interact with a biologically relevant bound ligand. Since it is not presently possible to clearly distinguish between catalytic, active, and binding site residues, using computational methods, the algorithms simply predict protein–ligand binding site residues. In CASP8, the top performing methods LEE [86] and 3DLigandSite [87] used a similar prediction strategy, combining information from homology models along with the templates used to construct the models that contained biologically-relevant bound ligands. In CASP9 [75] and CASP10 [76], successful methods for the prediction of protein–ligand binding sites built upon and further refined this template-based approach. 

Following on from CASP10 [76], it was decided to move the FN prediction category to a continuous assessment strategy, due to the lack of available targets containing bound biologically-relevant ligands during the short three month CASP prediction period. Hence, the CASP FN category moved to the CAMEO continuous assessment project [65]. The move to fully automated assessment resulted in a change of prediction format, with the additional prediction of which ligand category (I—Ion, O—Organic, N—Nucleotide, and P—Peptide) a protein may bind. Participating servers must also provide a *p*-value representing the likelihood that each residue (or atom) binds a ligand in each category. The CAMEO assessment runs weekly on structures containing biologically-relevant ligands using target sequences of structures that are on hold for release by the Protein Data Bank (PDB) [65]. The CAMEO project provides a better picture of how each method performs on a large and diverse dataset, containing a wide variety of proteins bound to a wide variety of ligands. 

Complementary to CAMEO and CASP is the CAFA [85] experiment, which has also been a major driver for the development of function prediction methods. The goal of CAFA is to functionally annotate proteins on a large scale using GO terms [70,71]. The CAFA1 dataset contained >48,000 proteins as of October 2010, for which predictions were made. Following the prediction season, methods were evaluated on 866 of the proteins, which had acquired annotations over the eleven months following the close of the prediction season. Methods that compete in CAFA [85] include a large number of the methods described in the proceeding sections, comprising sequence-based methods, structure-based methods and combinations of both. 

## 6. The Application of *in Silico* Protein–Ligand Binding Site Prediction Methods: Impact on *in Vitro* Studies

In addition to the theoretical and computational uses of protein–ligand binding site prediction algorithms previously highlighted, methods for the prediction of protein–ligand binding sites have been used in numerous *in silico*/*in vitro* studies. These studies have focused on a wide range of subjects as diverse as calcium-binding proteins [88], olfactory proteins [89], the CollagenQ protein–COLQ [90], human PE5 proteins [91], barley powdery mildew proteins [92,93], and spider mite glutathione S-transferases [94], which have led to biological findings of relevance to the study of health and disease and to food security [88,89,90,91,92,93,94,95].

We firstly describe a number of case studies from research projects investigating proteins implicated in health and disease. The first study [88] analysed a large number of calcium-binding proteins present in biological systems on a genome-wide scale, termed: calciomics. As calcium impacts every aspect of cellular life, Ca^2+^ binding proteins can be implicated in a wide range of diseases, thus this *in silico* study investigates their potential roles [88]. Another *in silico* proteome-wide study, this time on PE5 proteins (plasma membrane transporters and receptors) from the human proteome, was undertaken by Dong *et al.* to correct misannotations of these highly misannotated proteins [91]. Furthermore, Don and Riniker undertook *in silico* analysis of olfactory receptor proteins, members of the G-protein coupled receptor (GPCR) family, to enable the future design of therapeutics targeting olfactory-related and GPCR-related diseases [89]. In addition, Arredondo *et al.* combined modelling and the prediction of protein–ligand binding sites with *in vitro* studies to investigate a number of COLQ mutants and determine their mode of action [90]. These COLQ mutants cause human deficiency of endplate acetylcholinesterase, which results in the impairment of the interaction of COLQ with the basal lamina. This leads to a reduction in the duration of synaptic activation, which can lead to synaptic-related diseases.

Focusing on projects that have implications on food security, we highlight a study on the barley powdery mildew proteome [92]. This research involved the combination of proteogenomic along with structural and functional (protein–ligand binding sites and binding site residues) predictions, in order to investigate the pathogenic properties of barley powdery mildew. Basically, IntFOLD [63,64] was used to construct models for the entire proteome, which were validated utilizing ModFOLD3 [63]. Subsequently, FunFOLD [4] was used to predict protein–ligand binding sites for these models. This resulted in interesting conclusions about the *Blumera graminis* f.sp*. hordei* proteome. Firstly, the proteins are structurally diverse and remotely homologous to known proteins, potentially containing novel folds, as it was only possible to model six proteins with a model quality score above 0.4. Secondly, FunFOLD was able to help in the assignment of functionality for these six proteins, all were carbohydrate-binding and probably glycosyl hydrolases. Moreover, this putative functionality was experimentally elucidated, highlighting the utility of protein–ligand interaction methods to aid functional elucidation [92]. An additional study with relevance to food security from Pavlidi *et al.* [94] involves the functional characterization of a particular glutathione S-transferase, which may enable the two-spotted spider mite (*Tetranychus urticae*) to have acaricide/insecticide resistance. *Tetranychus urticae* has been shown to be one of the most damaging agricultural pests globally. The spider mite has three glutathione S-transferase enzymes; TuGSTd10, TuGSTd14, and TuGSTm09. Subsequently, assays determined that TuGSTd14 was the glutathione S-transferase involved in the acaricide/insecticide resistance. The structure of TuGSTd14 was predicted using IntFOLD [63,64] and protein–ligand binding site residues predicted using FunFOLD [3]. These *in silico* results were utilized to determine the key structural characteristics, including residues that were involved in the substrate binding specificity [94].

The studies described above, on proteins related to health and disease [88,89,90,91] in addition to food security [92,93,94] highlight the utility of protein–ligand binding site prediction methods, which can contribute to the interpretation of the function and the biochemical interactions of key proteins and enzymes, impacting our ability to tackle urgent global problems. 

## 7. Conclusions

A large number of predictive methods are available to predict and analyse protein–ligand binding sites. These methods incorporate different approaches, providing numerous different data types ranging from lists of ligand binding site residues, 3D atomic coordinates of ligand binding sites, lists of putative binding ligands, EC, and GO terms. The results produced by these *in silico* methods can be useful to generate new hypotheses and drive further experiments that can impact on major challenges in biology.

## References

[1] Roche D.B., Buenavista M.T., McGuffin L.J. (2012). FunFOLDQA: A quality assessment tool for protein–ligand binding site residue predictions. PLoS ONE.

[2] Roche D.B., Buenavista M.T., McGuffin L.J., Roberts G.C.K. (2012). Predicting protein structures and structural annotation of proteomes. Encyclopedia of Biophysics.

[3] Roche D.B., Buenavista M.T., McGuffin L.J. (2013). The FunFOLD2 server for the prediction of protein–ligand interactions. Nucleic Acids Res..

[4] Roche D.B., Tetchner S.J., McGuffin L.J. (2011). FunFOLD: An improved automated method for the prediction of ligand binding residues using 3D models of proteins. BMC Bioinforma..

[5] Rang H.P., Ritter J.M., Flower R.J., Henderson G. (2015). Rang and dale's pharmacology.

[6] Walsh A.A., Szklarz G.D., Scott E.E. (2013). Human cytochrome P450 1A1 structure and utility in understanding drug and xenobiotic metabolism. J. Biol. Chem..

[7] Yang K.H., Lee J.H., Lee M.G. (2008). Effects of CYP inducers and inhibitors on the pharmacokinetics of intravenous theophylline in rats: Involvement of CYP1A1/2 in the formation of 1,3-DMU. J. Pharm. Pharmacol..

[8] Lopez G., Ezkurdia I., Tress M.L. (2009). Assessment of ligand binding residue predictions in CASP8. Proteins.

[9] Kauffman C., Karypis G., Rangwala H., Karypis G. (2010). Ligand-binding residue prediction. Introduction to protein structure prediction: Methods and algorithms.

[10] Yuriev E., Holien J., Ramsland P.A. (2015). Improvements, trends, and new ideas in molecular docking: 2012–2013 in review. J. Mol. Recognit..

[11] Ye K., Feenstra K.A., Heringa J., Ijzerman A.P., Marchiori E. (2008). Multi-relief: A method to recognize specificity determining residues from multiple sequence alignments using a machine-learning approach for feature weighting. Bioinformatics.

[12] Yu D.-J., Hu J., Yang J., Shen H.-B., Tang J., Yang J.-Y. (2013). Designing template-free predictor for targeting protein–ligand binding sites with classifier ensemble and spatial clustering. IEEE/ACM Trans. Comput. Biol. Bioinform..

[13] Chen P., Huang J.H.Z., Gao X. (2014). Ligandrfs: Random forest ensemble to identify ligand-binding residues from sequence information alone. BMC Bioinforma..

[14] Yu D.J., Hu J., Li Q.M., Tang Z.M., Yang J.Y., Shen H.B. (2015). Constructing query-driven dynamic machine learning model with application to protein–ligand binding sites prediction. IEEE Trans. Nanobiosci..

[15] Sankararaman S., Kolaczkowski B., Sjolander K. (2009). Intrepid: A web server for prediction of functionally important residues by evolutionary analysis. Nucleic Acids Res..

[16] Sankararaman S., Sha F., Kirsch J.F., Jordan M.I., Sjolander K. (2010). Active site prediction using evolutionary and structural information. Bioinformatics.

[17] Ashkenazy H., Erez E., Martz E., Pupko T., Ben-Tal N. (2010). Consurf 2010: Calculating evolutionary conservation in sequence and structure of proteins and nucleic acids. Nucleic Acids Res..

[18] Wass M.N., Sternberg M.J. (2008). Confunc—functional annotation in the twilight zone. Bioinformatics.

[19] Wierschin T., Wang K., Welter M., Waack S., Stanke M. (2015). Combining features in a graphical model to predict protein binding sites. Proteins.

[20] Kononenko I. (1994). Estimating attributes: Analysis and extensions of relief. Proceedings of the European Conference on Machine Learning.

[21] Kawashima S., Pokarowski P., Pokarowska M., Kolinski A., Katayama T., Kanehisa M. (2008). AAindex: Amino acid index database, progress report 2008. Nucleic Acids Res..

[22] Altschul S.F., Madden T.L., Schaffer A.A., Zhang J., Zhang Z., Miller W., Lipman D.J. (1997). Gapped BLAST and PSI-BLAST: A new generation of protein database search programs. Nucleic Acids Res..

[23] UniProt C. (2015). UniProt: A hub for protein information. Nucleic Acids Res..

[24] McGuffin L.J., Bryson K., Jones D.T. (2000). The PSIPRED protein structure prediction server. Bioinformatics.

[25] Brylinski M., Skolnick J. (2008). A threading-based method (FINDSITE) for ligand-binding site prediction and functional annotation. Proc. Natl. Acad. Sci. USA.

[26] Fuller J.C., Martinez M., Henrich S., Stank A., Richter S., Wade R.C. (2015). Ligdig: A web server for querying ligand-protein interactions. Bioinformatics.

[27] Xie Z.R., Liu C.K., Hsiao F.C., Yao A., Hwang M.J. (2013). LISE: A server using ligand-interacting and site-enriched protein triangles for prediction of ligand-binding sites. Nucleic Acids Res..

[28] Zhu X., Xiong Y., Kihara D. (2015). Large-scale binding ligand prediction by improved patch-based method patch-surfer2.0. Bioinformatics.

[29] Spitzer R., Cleves A.E., Jain A.N. (2011). Surface-based protein binding pocket similarity. Proteins.

[30] Erdin S., Ward R.M., Venner E., Lichtarge O. (2010). Evolutionary trace annotation of protein function in the structural proteome. J. Mol. Biol..

[31] Krivak R., Hoksza D. (2015). Improving protein–ligand binding site prediction accuracy by classification of inner pocket points using local features. J. Cheminform..

[32] Kokubo H., Tanaka T., Okamoto Y. (2013). Two-dimensional replica-exchange method for predicting protein–ligand binding structures. J. Comput. Chem..

[33] Chang L., Ishikawa T., Kuwata K., Takada S. (2013). Protein-specific force field derived from the fragment molecular orbital method can improve protein–ligand binding interactions. J. Comput. Chem..

[34] Estrada T., Zhang B., Cicotti P., Armen R.S., Taufer M. (2012). A scalable and accurate method for classifying protein–ligand binding geometries using a mapreduce approach. Comput. Biol. Med..

[35] Desaphy J., Raimbaud E., Ducrot P., Rognan D. (2013). Encoding protein–ligand interaction patterns in fingerprints and graphs. J. Chem. Inf. Model..

[36] Santos J.C.A., Nassif H., Page D., Muggleton S.H., Sternberg M.J.E. (2012). Automated identification of protein–ligand interaction features using inductive logic programming: A hexose binding case study. BMC Bioinform..

[37] Jacob L., Vert J.P. (2008). Protein-ligand interaction prediction: An improved chemogenomics approach. Bioinformatics.

[38] Seifert M.H., Schmitt F., Herz T., Kramer B. (2004). Propose: A docking engine based on a fully configurable protein–ligand interaction model. J. Mol. Model..

[39] Das S., Sillitoe I., Lee D., Lees J.G., Dawson N.L., Ward J., Orengo C.A. (2015). Cath funfhmmer web server: Protein functional annotations using functional family assignments. Nucleic Acids Res..

[40] He W., Liang Z., Teng M., Niu L. (2015). Mfasd: A structure-based algorithm for discriminating different types of metal-binding sites. Bioinformatics.

[41] Konc J., Janezic D. (2012). Probis-2012: Web server and web services for detection of structurally similar binding sites in proteins. Nucleic Acids Res..

[42] Konc J., Janezic D. (2010). Probis algorithm for detection of structurally similar protein binding sites by local structural alignment. Bioinformatics.

[43] Krotzky T., Fober T., Hullermeier E., Klebe G. (2014). Extended graph-based models for enhanced similarity search in cavbase. IEEE/ACM Trans. Comput. Biol. Bioinform..

[44] Schmitt S., Kuhn D., Klebe G. (2002). A new method to detect related function among proteins independent of sequence and fold homology. J. Mol. Biol..

[45] Hernandez M., Ghersi D., Sanchez R. (2009). Sitehound-web: A server for ligand binding site identification in protein structures. Nucleic Acids Res..

[46] Amari S., Aizawa M., Zhang J., Fukuzawa K., Mochizuki Y., Iwasawa Y., Nakata K., Chuman H., Nakano T. (2006). Viscana: Visualized cluster analysis of protein–ligand interaction based on the ab initio fragment molecular orbital method for virtual ligand screening. J. Chem. Inf. Model..

[47] Lin Y., Yoo S., Sanchez R. (2012). Sitecomp: A server for ligand binding site analysis in protein structures. Bioinformatics.

[48] Kozakov D., Grove L.E., Hall D.R., Bohnuud T., Mottarella S.E., Luo L., Xia B., Beglov D., Vajda S. (2015). The FTMap family of web servers for determining and characterizing ligand-binding hot spots of proteins. Nat. Protoc..

[49] Yang J., Roy A., Zhang Y. (2013). Protein-ligand binding site recognition using complementary binding-specific substructure comparison and sequence profile alignment. Bioinformatics.

[50] Roy A., Yang J., Zhang Y. (2012). Cofactor: An accurate comparative algorithm for structure-based protein function annotation. Nucleic Acids Res..

[51] Heo L., Shin W.H., Lee M.S., Seok C. (2014). GalaxySite: Ligand-binding-site prediction by using molecular docking. Nucleic Acids Res..

[52] Izidoro S.C., de Melo-Minardi R.C., Pappa G.L. (2014). GASS: Identifying enzyme active sites with genetic algorithms. Bioinformatics.

[53] Guo Z., Li B., Cheng L.T., Zhou S., McCammon J.A., Che J. (2015). Identification of protein–ligand binding sites by the level-set variational implicit-solvent approach. J. Chem. Theory Comput..

[54] Salentin S., Schreiber S., Haupt V.J., Adasme M.F., Schroeder M. (2015). Plip: Fully automated protein–ligand interaction profiler. Nucleic Acids Res..

[55] Huang B., Schroeder M. (2006). Ligsitecsc: Predicting ligand binding sites using the connolly surface and degree of conservation. BMC Struct. Biol..

[56] Andersson C.D., Chen B.Y., Linusson A. (2010). Mapping of ligand-binding cavities in proteins. Proteins.

[57] Roche D.B., McGuffin L.J., Stoddard B., Baker D. (2015). *In silico* identification and characterization of protein–ligand binding sites, methods in molecular biology. Structure based and computational design of ligand binding proteins.

[58] Hastings J., de Matos P., Dekker A., Ennis M., Harsha B., Kale N., Muthukrishnan V., Owen G., Turner S., Williams M. (2013). The ChEBi reference database and ontology for biologically relevant chemistry: Enhancements for 2013. Nucleic Acids Res..

[59] Berman H.M., Westbrook J., Feng Z., Gilliland G., Bhat T.N., Weissig H., Shindyalov I.N., Bourne P.E. (2000). The protein data bank. Nucleic Acids Res..

[60] Okuda S., Yamada T., Hamajima M., Itoh M., Katayama T., Bork P., Goto S., Kanehisa M. (2008). KEGG Atlas mapping for global analysis of metabolic pathways. Nucleic Acids Res..

[61] Xie Z.R., Hwang M.J. (2010). An interaction-motif-based scoring function for protein–ligand docking. BMC Bioinform..

[62] Petrey D., Chen T.S., Deng L., Garzon J.I., Hwang H., Lasso G., Lee H., Silkov A., Honig B. (2015). Template-based prediction of protein function. Curr. Opin. Struct. Biol..

[63] Roche D.B., Buenavista M.T., Tetchner S.J., McGuffin L.J. (2011). The intfold server: An integrated web resource for protein fold recognition, 3D model quality assessment, intrinsic disorder prediction, domain prediction and ligand binding site prediction. Nucleic Acids Res..

[64] McGuffin L.J., Atkins J.D., Salehe B.R., Shuid A.N., Roche D.B. (2015). IntFOLD: An integrated server for modelling protein structures and functions from amino acid sequences. Nucleic Acids Res..

[65] Haas J., Roth S., Arnold K., Kiefer F., Schmidt T., Bordoli L., Schwede T. (2013). The protein model portal—A comprehensive resource for protein structure and model information. Database.

[66] Zhang Y., Skolnick J. (2005). Tm-align: A protein structure alignment algorithm based on the TM-score. Nucleic Acids Res..

[67] Yang J., Roy A., Zhang Y. (2013). Biolip: A semi-manually curated database for biologically relevant ligand-protein interactions. Nucleic Acids Res..

[68] Roche D.B., Tetchner S.J., McGuffin L.J. (2010). The binding-site distance test score: A robust method for the assessment of predicted protein binding sites. Bioinformatics.

[69] Matthews B.W. (1975). Comparison of the predicted and observed secondary structure of T4 phage lysozyme. Biochim. Biophys. Acta..

[70] Ashburner M., Ball C.A., Blake J.A., Botstein D., Butler H., Cherry J.M., Davis A.P., Dolinski K., Dwight S.S., Eppig J.T. (2000). Gene ontology: Tool for the unification of biology. Nat. Genet..

[71] Gene Ontology Consortium (2015). Gene ontology consortium: Going forward. Nucleic Acids Res..

[72] Capra J.A., Laskowski R.A., Thornton J.M., Singh M., Funkhouser T.A. (2009). Predicting protein ligand binding sites by combining evolutionary sequence conservation and 3D structure. PLoS Comput. Biol..

[73] Furnham N., Holliday G.L., de Beer T.A., Jacobsen J.O., Pearson W.R., Thornton J.M. (2014). The catalytic site atlas 2.0: Cataloging catalytic sites and residues identified in enzymes. Nucleic Acids Res..

[74] Talavera D., Laskowski R.A., Thornton J.M. (2009). Wssas: A web service for the annotation of functional residues through structural homologues. Bioinformatics.

[75] Schmidt T., Haas J., Gallo Cassarino T., Schwede T. (2011). Assessment of ligand-binding residue predictions in casp9. Proteins.

[76] Gallo Cassarino T., Bordoli L., Schwede T. (2014). Assessment of ligand binding site predictions in CASP10. Proteins.

[77] McDonald A.G., Tipton K.F. (2014). Fifty-five years of enzyme classification: Advances and difficulties. FEBS J..

[78] Piovesan D., Giollo M., Leonardi E., Ferrari C., Tosatto S.C. (2015). Inga: Protein function prediction combining interaction networks, domain assignments and sequence similarity. Nucleic Acids Res..

[79] Gerlt J.A., Bouvier J.T., Davidson D.B., Imker H.J., Sadkhin B., Slater D.R., Whalen K.L. (2015). Enzyme function initiative-enzyme similarity tool (EFI-EST): A web tool for generating protein sequence similarity networks. Biochim. Biophys. Acta.

[80] Sahraeian S.M., Luo K.R., Brenner S.E. (2015). Sifter search: A web server for accurate phylogeny-based protein function prediction. Nucleic Acids Res..

[81] Gundersen G.W., Jones M.R., Rouillard A.D., Kou Y., Monteiro C.D., Feldmann A.S., Hu K.S., Ma’ayan A. (2015). Geo2enrichr: Browser extension and server app to extract gene sets from geo and analyze them for biological functions. Bioinformatics.

[82] Koskinen P., Toronen P., Nokso-Koivisto J., Holm L. (2015). Pannzer: High-throughput functional annotation of uncharacterized proteins in an error-prone environment. Bioinformatics.

[83] Yu G., Zhu H., Domeniconi C. (2015). Predicting protein functions using incomplete hierarchical labels. BMC Bioinform..

[84] Wang T., Mori H., Zhang C., Kurokawa K., Xing X.H., Yamada T. (2015). Domsign: A top-down annotation pipeline to enlarge enzyme space in the protein universe. BMC Bioinforma..

[85] Radivojac P., Clark W.T., Oron T.R., Schnoes A.M., Wittkop T., Sokolov A., Graim K., Funk C., Verspoor K., Ben-Hur A. (2013). A large-scale evaluation of computational protein function prediction. Nat. Methods.

[86] Oh M., Joo K., Lee J. (2009). Protein-binding site prediction based on three-dimensional protein modeling. Proteins.

[87] Wass M.N., Kelley L.A., Sternberg M.J. (2010). 3DLigandsite: Predicting ligand-binding sites using similar structures. Nucleic Acids Res..

[88] Zhou Y., Xue S., Yang J.J. (2013). Calciomics: Integrative studies of Ca^2+^-binding proteins and their interactomes in biological systems. Metallomics.

[89] Don C.G., Riniker S. (2014). Scents and sense: *In silico* perspectives on olfactory receptors. J. Comput. Chem..

[90] Arredondo J., Lara M., Ng F., Gochez D.A., Lee D.C., Logia S.P., Nguyen J., Maselli R.A. (2014). Cooh-terminal collagen Q (COLQ) mutants causing human deficiency of endplate acetylcholinesterase impair the interaction of ColQ with proteins of the basal lamina. Hum. Genet..

[91] Dong Q., Menon R., Omenn G.S., Zhang Y. (2015). Structural bioinformatics inspection of nextprot PE5 proteins in the human proteome. J. Proteome Res..

[92] Bindschedler L.V., McGuffin L.J., Burgis T.A., Spanu P.D., Cramer R. (2011). Proteogenomics and *in silico* structural and functional annotation of the barley powdery mildew *blumeria graminis* f. sp. *hordei*. Methods.

[93] Pedersen C., Ver Loren van Themaat E., McGuffin L.J., Abbott J.C., Burgis T.A., Barton G., Bindschedler L.V., Lu X., Maekawa T., Wessling R. (2012). Structure and evolution of barley powdery mildew effector candidates. BMC Genomics.

[94] Pavlidi N., Tseliou V., Riga M., Nauen R., Van Leeuwen T., Labrou N.E., Vontas J. (2015). Functional characterization of glutathione S-transferases associated with insecticide resistance in *Tetranychus urticae*. Pestic. Biochem. Physiol..

[95] Taylor T.B., Mulley G., Dills A.H., Alsohim A.S., McGuffin L.J., Studholme D.J., Silby M.W., Brockhurst M.A., Johnson L.J., Jackson R.W. (2015). Evolutionary resurrection of flagellar motility via rewiring of the nitrogen regulation system. Science.

